# Caffeic Acid Inhibits the Formation of 7-Carboxyheptyl Radicals from Oleic Acid under Flavin Mononucleotide Photosensitization by Scavenging Singlet Oxygen and Quenching the Excited State of Flavin Mononucleotide

**DOI:** 10.3390/molecules190812486

**Published:** 2014-08-18

**Authors:** Marie Asano, Hideo Iwahashi

**Affiliations:** Department of Chemistry, Wakayama Medical University, 580 Mikazura, Wakayama 641-0011, Japan; E-Mail: asano@wakayama-med.ac.jp

**Keywords:** caffeic acid, FMN, oleic acid, photosensitization reaction, 7-carboxyheptyl radical, singlet oxygen, phenol, spin trapping, HPLC-ESR

## Abstract

We examined the effects of caffeic acid (CA) and related compounds on 7-carboxyheptyl radical formation. This analysis was performed using a standard D_2_O reaction mixture containing 4.3 mM oleic acid, 25 μM flavin mononucleotide (FMN), 160 mM phosphate buffer (pH 7.4), 10 mM cholic acid, 100 mM α-(4-pyridyl-1-oxide)-*N*-*tert*-butylnitrone, and 1 mM Fe(SO_4_)_2_(NH_4_)_2_ during irradiation with 7.8 J/cm^2^ at 436 nm. 7-Carboxyheptyl radical formation was inhibited by CA, catechol, gallic acid, chlorogenic acid, ferulic acid, noradrenalin, 2-hydroxybenzoic acid, 3-hydroxybenzoic acid, and 4-hydroxybenzoic acid. Quinic acid, benzoic acid, and *p*-anisic acid had no effect on radical formation. These results suggest that a phenol moiety is essential for these inhibitory effects. The fluorescence intensity of FMN decreased by 69% ± 2% after CA addition, suggesting that CA quenches the singlet excited state of FMN. When 1 mM CA was added to a standard reaction mixture containing 25 μM FMN, 140 mM phosphate buffer (pH 7.4), and 10 mM 4-oxo-2,2,6,6-tetramethylpiperidine, the electron spin resonance signal of 4-oxo-2,2,6,6-tetramethylpiperidinooxy disappeared. This finding suggests that singlet oxygen was scavenged completely by CA. Therefore, CA appears to inhibit 7-carboxyheptyl radical formation by scavenging singlet oxygen and quenching the excited state of FMN.

## 1. Introduction

Oxidative stress can result from many different unfavorable environmental factors such as ultraviolet (UV) light [1,2], smoking [3], and air pollutants [4,5]. Oxidative stress occurs when levels of reactive oxygen species (ROS), including superoxide anion (O_2_^−•^), hydrogen peroxide (H_2_O_2_), and hydroxyl radicals (^•^OH), are elevated. These species can target lipid-rich membranes, cellular DNA, and proteins to exert an array of toxic effects.

Singlet oxygen (^1^O_2_) is a form of ROS that can be generated in biological systems. The reactions of singlet oxygen have drawn much attention due to the ease with which singlet oxygen can be generated during conventional photosensitized reactions. The type-II reaction of a photosensitizer transfers its energy to a ground state oxygen molecule to form ^1^O_2_ [6], which is a very powerful oxidant with a relatively long lifetime and can react with many intracellular macromolecules, including lipids [7].

Polyphenols exist ubiquitously in Nature. As such, many beverages and foods are major sources of polyphenols that can protect against oxidative stress via their antioxidant activity. Several studies have demonstrated that polyphenols scavenge free radicals via hydrogen donation and aromatic hydroxylation [8,9,10]. However, other studies have suggested that polyphenols inhibit the formation of free radicals and the propagation of free radical reactions by chelating transition metal ions [11,12,13].

Coffee is one of the most popular beverages worldwide. Many studies have suggested that coffee exerts beneficial health effects. For example, coffee consumption may reduce the risk of type 2 diabetes [14,15,16], cardiovascular and inflammatory diseases [17], and Parkinson’s and Alzheimer’s diseases [18]. Coffee is an extremely rich source of phenolic acids, including aromatic hydroxycinnamic acids such as caffeic acid [3,4-dihydroxycinnamic acid (CA)]). CA is found in coffee mainly as chlorogenic acid (5-*O*-caffeoylquinic acid). The chlorogenic acid content in an average sized cup of coffee (200 mL) ranges from 70 to 350 mg, which provides ~35–175 mg of CA [19]. CA is also found in fruits and vegetables [20].

Ferulic acid and CA provided protective effects on UVA-mediated matrix metalloprotease-1 induction in immortalized human keratinocyte cells possibly through restoration of antioxidant defense system at the celluar and molecular level [21]. CA also inhibits UVB-induced cyclooxygenase 2 expression and the subsequent prostaglandin E2 production in mouse skin epidermal cells and mouse skin *in vivo* [22]. Therefore, chlorogenic acid and CA are widely known as antioxidants [23], including functions as O_2_^−•^ and OH^•^ scavengers [24,25]. In addition, CA effectively suppresses ROS generation in the skin of animals after exposure to UVA [26]. The antioxidant activities of CA could also play a role in protecting against UVB oxidative damage in human erythrocytes and LDL [27]. It has been reported that CA inhibits the formation of radicals by chelating ferrous ions [28,29].

In this study, we examined the antioxidant properties of CA and related compounds on the formation of oleic acid-derived radicals during flavin mononucleotide (FMN) photosensitization.

## 2. Results and Discussion

### 2.1. Electron Spin Resonance (ESR) Analysis of the Standard Reaction Mixture (I) with CA

**Figure 1:**
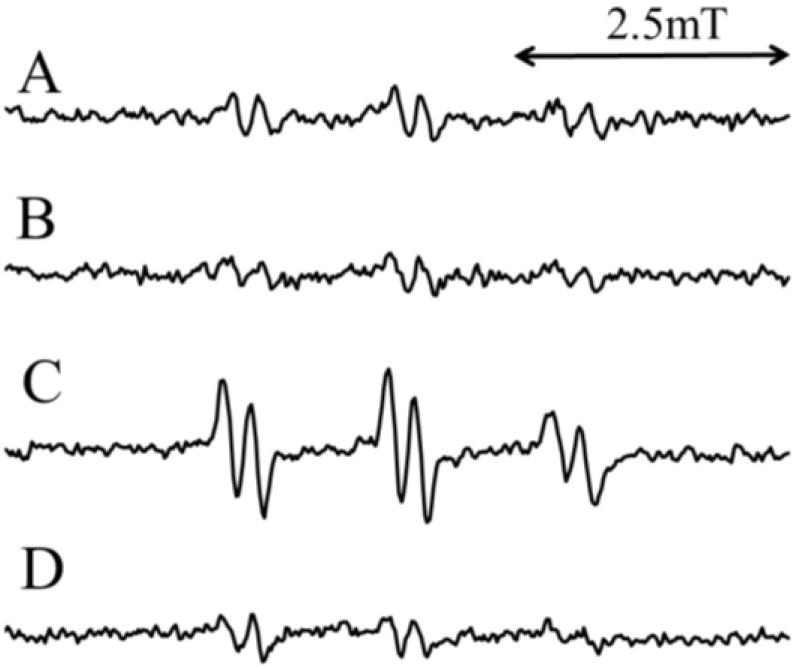
ESR spectra of the standard reaction mixture (I) irradiated with visible light at 436 nm. (**A**) Standard H_2_O reaction mixture (I); (**B**) Standard H_2_O reaction mixture (I) with 2 mM CA; (**C**) Standard D_2_O reaction mixture (I); (**D**) Standard D_2_O reaction mixture (I) with 2 mM CA. The reaction and ESR conditions are described in the Experimental Section.

Weak ESR signals were observed when the standard H_2_O reaction mixture (I) was assayed (Figure 1A), suggesting that free radicals were formed in the standard H_2_O reaction mixture (I). The free radicals were previously identified as α-(4-pyridyl-1-oxide)-*N*-*tert*-butylnitrone (4-POBN)/7-carboxyheptyl radical adducts [30]. After the addition of 2 mM CA to the standard H_2_O reaction mixture (I), the peak height of the ESR signals decreased slightly (78.7% ± 7.2% of the standard H_2_O reaction mixture) (Figure 1B). Previously we have shown that singlet oxygen may be involved in the formation of the 4-POBN/7-carboxyheptyl radical adduct in the standard H_2_O reaction mixture (I) [30]. To improve the signal:noise ratio of the ESR signal, the reaction was performed in D_2_O, since D_2_O increases the half-life of singlet oxygen [6]. Prominent ESR signals (α^N^ = 1.58 mT and α^Hβ^ = 0.26 mT) (Figure 1C) were observed for the standard D_2_O reaction mixture (I) [368% ± 51% of the standard H2O reaction mixture (I)]. The addition of 2 mM CA to the standard D_2_O reaction mixture (I) resulted in obviously decreased peak heights of the ESR signals (Figure 1D) [27.8% ± 1.4% of the standard D_2_O reaction mixture (I)].

### 2.2. High Performance Liquid Chromatography (HPLC)-ESR Analyses of the Standard D_2_O Reaction Mixture (I) with CA

**Figure 2:**
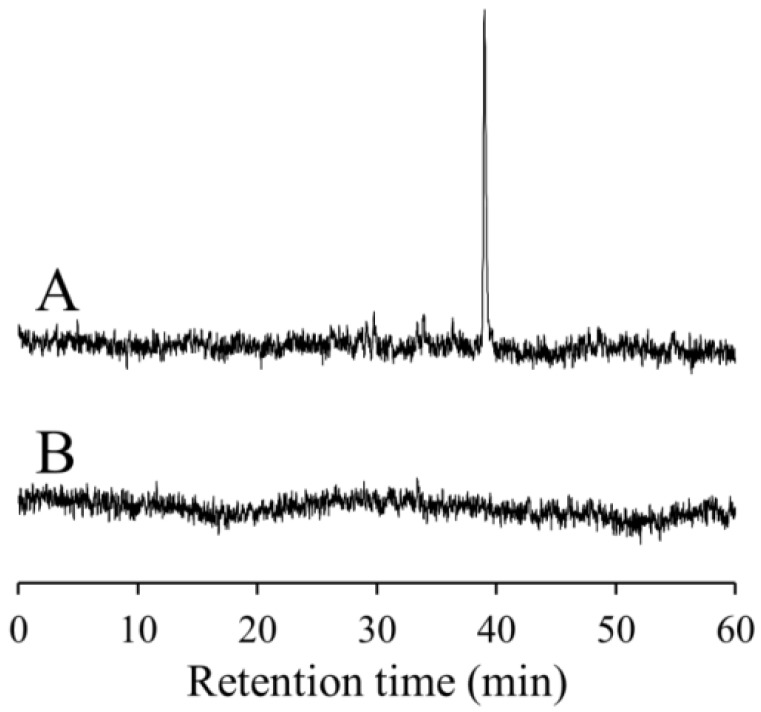
High performance liquid chromatography (HPLC)-electron spin resonance (HPLC-ESR) analyses of the standard D_2_O reaction mixture (I). The reaction and HPLC-ESR conditions are described in the Experimental Section. The total reaction volume was 1.5 mL. HPLC-ESR analyses for (**A**) the standard D_2_O reaction mixture (I); and (**B**) the standard D_2_O reaction mixture (I) with 2 mM CA.

HPLC-ESR analysis of the standard D_2_O reaction mixture (I) revealed the presence of a predominant peak with a retention time of 39 min (Figure 2A). The peak was previously identified as 4-POBN/7-carboxyheptyl radical adduct [30]. The peak was not observed in standard D_2_O reaction mixtures (I) containing 2 mM CA, suggesting that CA inhibits formation of the peak radical (Figure 2B).

### 2.3. Effect of CA and Its Related Compounds on the Formation of Free Radicals

**Figure 3:**
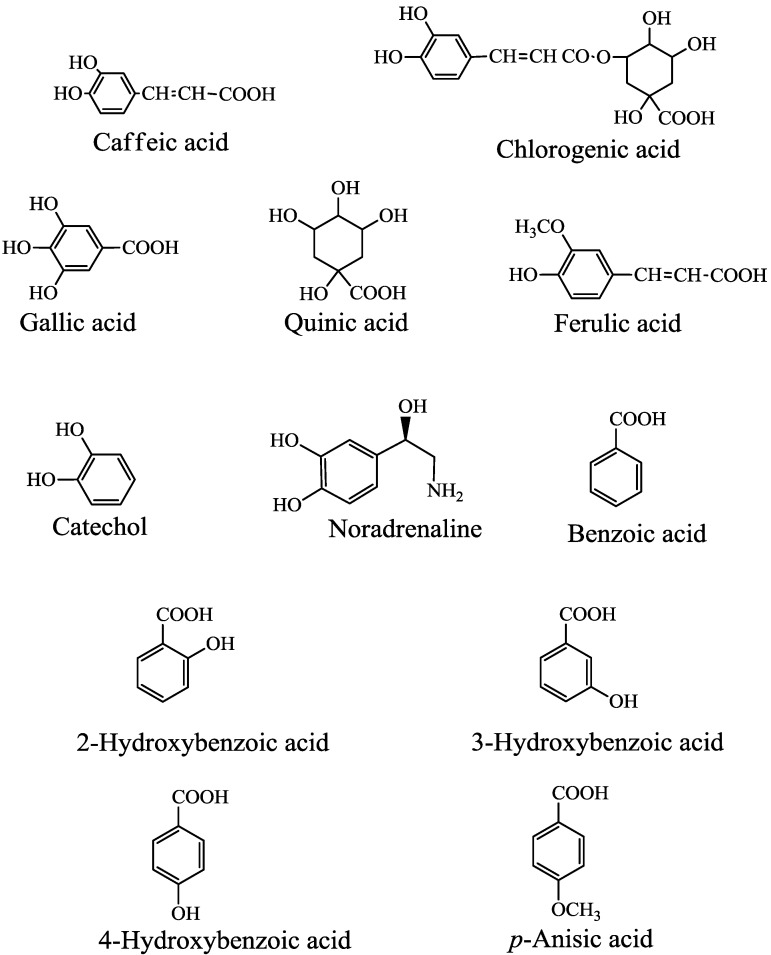
Chemical structures of caffeic acid and related compounds.

The effects of CA and its related compounds (Figure 3) on the formation of free radicals were assessed in standard D_2_O reaction mixture (I).

**Figure 4:**
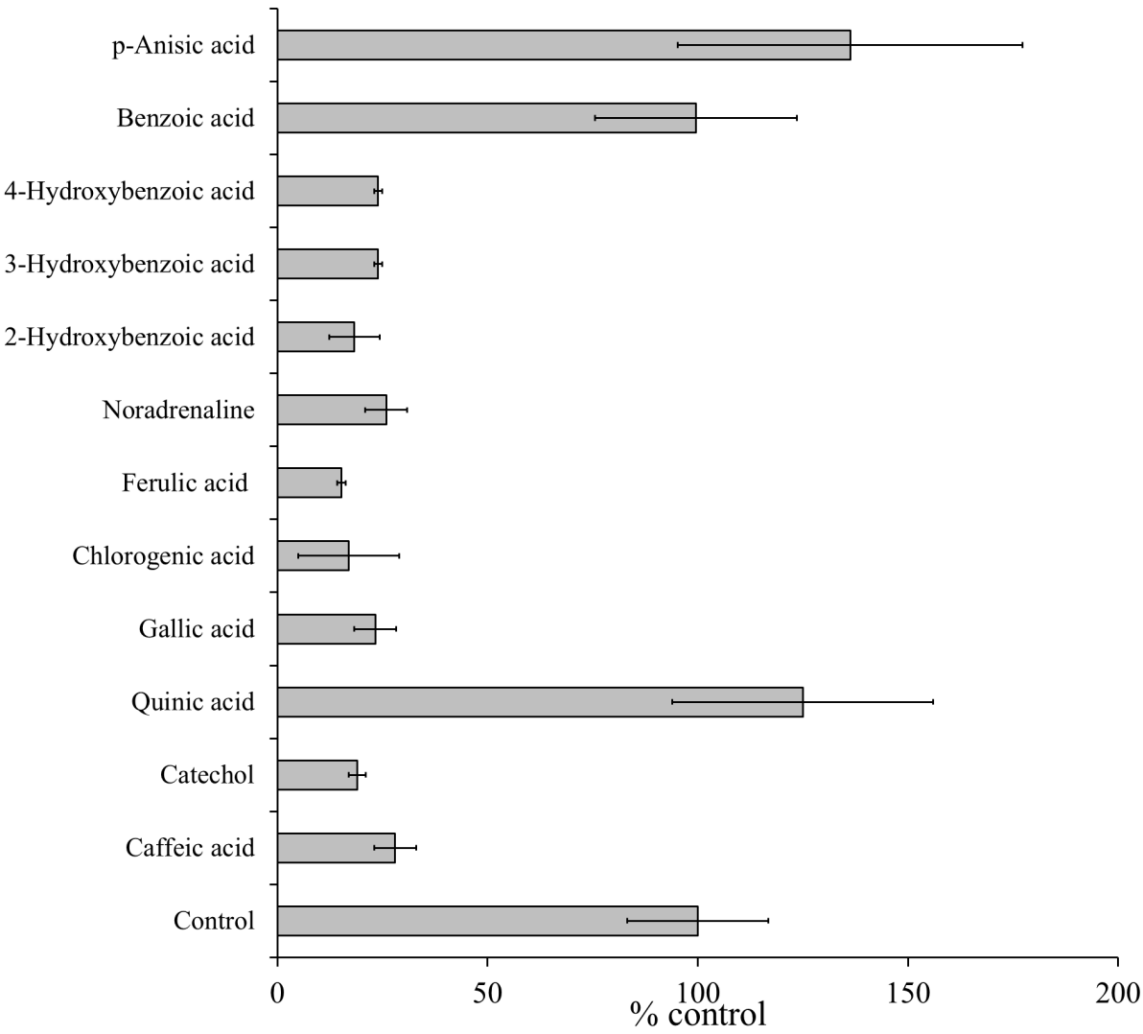
Effects of caffeic acid and related compounds on the formation of radicals in standard D_2_O reaction mixture (I) under visible light at 436 nm. ESR spectra were observed for the standard D_2_O reaction mixture (I) in the presence of 2 mM caffeic acid, catechol, gallic acid, chlorogenic acid, ferulic acid, noradrenalin, 2-hydroxybenzoic acid, 3-hydroxybenzoic acid, 4-hydroxybenzoic acid, quinic acid, benzoic acid, or *p*-anisic acid. The control value 100% represents the amount of 4-POBN radical adducts formed in the standard D_2_O reaction mixture (I). The respective values are the mean ± standard deviation of three determinations. The reaction and ESR conditions are as described in the Experimental Section.

The ESR spectra of the standard D_2_O reaction mixture (I) containing 2 mM CA (or its related compounds) were measured. The ESR peak height of the 4-POBN/free radical adducts decreased to 28% ± 5% (CA), 19% ± 2% (catechol), 23.3% ± 5% (gallic acid), 17% ± 12% (chlorogenic acid), 15.3% ± 1% (ferulic acid), 25.9% ± 5% (noradrenalin), 18.3% ± 6% (2-hydroxybenzoic acid), 24% ± 1% (3-hydroxybenzoic acid), and 24% ± 1% (4-hydroxybenzoic acid) of the peak in the standard D_2_O reaction mixtures (I). In contrast, quinic acid, benzoic acid, and *p*-anisic acid had no significant effect on free radical formation, with peaks of 125% ± 31% (quinic acid), 99.7% ± 24% (benzoic acid), and 136% ± 41% (*p*-anisic acid), respectively (Figure 4). These results suggested that the phenol moiety was essential for the inhibitory effects.

### 2.4. Iron Chelation by CA

**Figure 5:**
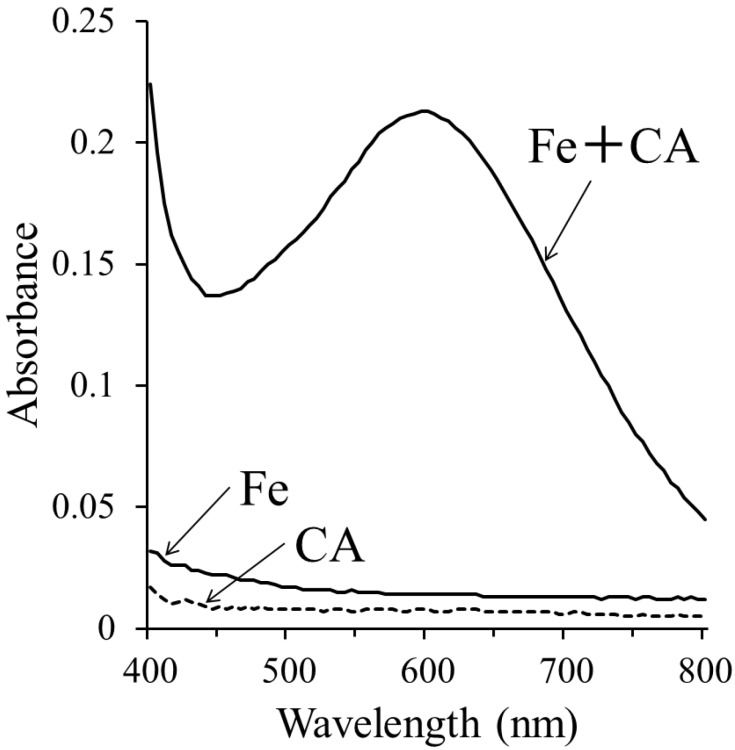
Visible absorption spectra of 1 mM Fe(SO_4_)_2_(NH_4_)_2_, 2 mM CA alone, or a combination of 1 mM Fe(SO_4_)_2_(NH_4_)_2_ and 2 mM CA. The conditions for the measurements are described in the Experimental Section.

CA has been previously reported to function as an O_2_^−•^ and OH^•^ scavenger [24,25] and an iron chelator [28,29]. To confirm that CA chelates iron ions, the visible absorption spectra were measured for the standard reaction mixture (IV), and a broad band with a λ_max_ of 597 nm was observed (Figure 5). The standard reaction mixture (IV) lacking 2 mM CA or 1 mM Fe(SO_4_)_2_(NH_4_)_2_ showed no prominent absorption in the visible region. This finding suggests that CA possibly chelates ferrous ions.

### 2.5. A Possible Reaction Path for the Formation of 7-Carboxyheptyl Radicals in the Standard D_2_O Reaction Mixture (I)

**Scheme 1:**
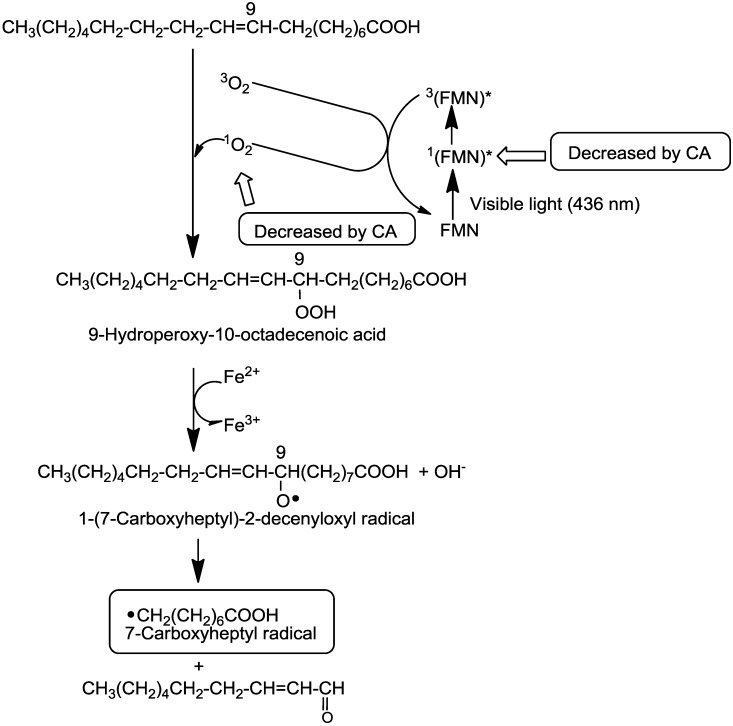
Possible inhibitory mechanisms of CA in the formation of the 7-carboxyheptyl radical in the standard D_2_O reaction mixture (I).

Previous studies have suggested a possible reaction path for the formation of 7-carboxyheptyl radicals (Scheme 1) [30]. The excited singlet state of FMN, ^1^(FMN)*, seems to participate in radical formation. This singlet state of FMN, which may be produced by irradiation with visible light at 436 nm, is possibly converted to the excited triplet state, ^3^(FMN)*, via intersystem crossing; it might then react with triplet oxygen, (^3^O_2_) to form ^1^O_2_ (Equation (1)) [31,32]:

^3^(FMN)* + ^3^O_2_ → FMN + ^1^O_2_ (1)

The reaction of oleic acid with ^1^O_2_ seems to form 9-hydroperoxy-10-octadecenoic acid through the ^1^O_2_ ene reaction [33]. Ferrous ions might then catalyze the decomposition of 9-hydroperoxy-10-octadecenoic acid. This reaction yields a radical intermediate LO^•^ (1-[7-carboxyheptyl]-2-decenyloxyl radical). The β-secession of LO^•^ could then result in the generation of the 7-carboxyheptyl radical [34].

**Figure 6:**
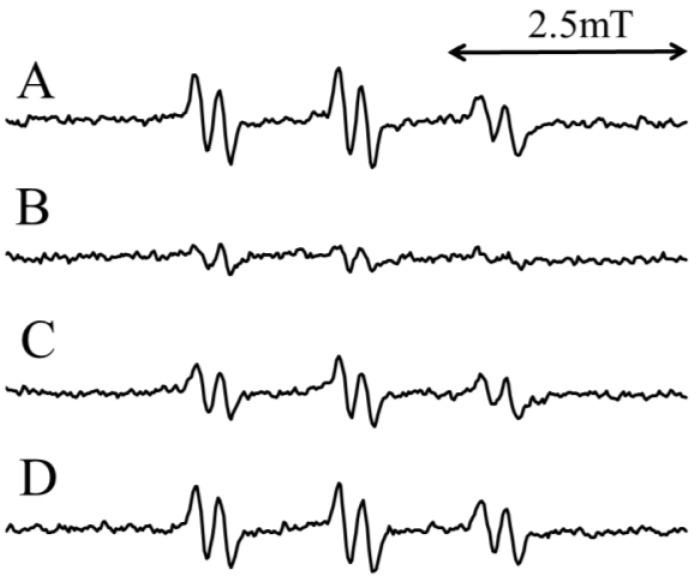
ESR spectra of the standard D_2_O reaction mixtures (I) and (II) irradiated with visible light at 436 nm. Reaction and ESR conditions are as described in the Experimental Section. (**A**) Standard D_2_O reaction mixture (I); (**B**) Standard D_2_O reaction mixture (I) with 2 mM CA, which was added before irradiation; (**C**) Standard D_2_O reaction mixture (II); (**D**) Standard D_2_O reaction mixture (II) with 1.7 mM CA, which was added after irradiation.

CA functions as an iron chelator [28,29], as shown in Figure 5. CA may inhibit the reaction from 9-hydroperoxy-10-octadecenoic acid to the 1-(7-carboxyheptyl)-2-decenyloxyl radical, because ferrous ions participate in the reaction. The ESR peak height decreased to 27.8% ± 1.4% of the standard D_2_O reaction mixtures (I) when CA was added prior to irradiation (Figure 6B). Conversely, when CA was added to the standard D_2_O reaction mixture (II) after irradiation, the ESR peak height increased to 153% ± 30.9% of the standard D_2_O reaction mixtures (II) (Figure 6D). Therefore, CA did not inhibit the conversion of 9-hydroperoxy-10-octadecenoic acid to 1-(7-carboxyheptyl)-2-decenyloxyl radicals. Similarly, CA did not scavenge 1-(7-carboxyheptyl)-2-decenyloxyl or 7-carboxyheptyl radicals.

### 2.6. Effects of CA on the Singlet Excited State of FMN and ^1^O_2_

**Figure 7:**
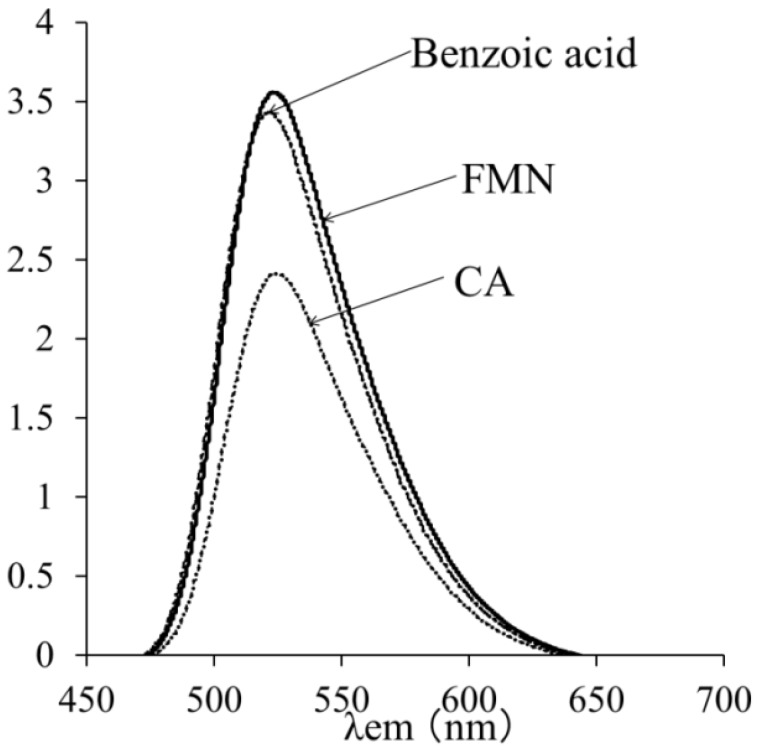
Fluorescence spectra of flavin mononucleotide alone or with 2.5 mM CA or 2.5 mM benzoic acid. The conditions for the measurements are as described in the Experimental Section.

To determine whether CA or benzoic acid quenched the singlet excited state of FMN, fluorescence spectra were measured for the standard reaction mixture (V). An intense fluorescence spectrum was observed for the standard reaction mixture (V) alone, and the addition of 2.5 mM benzoic acid had no effect on the fluorescence intensity. In contrast, the fluorescence intensity decreased by 69% ± 2% in the presence of 2.5 mM CA, suggesting that CA quenched the singlet excited state of FMN (Figure 7). It has been reported that some phenolic compounds quench the riboflavin triplet state [35].

**Figure 8:**
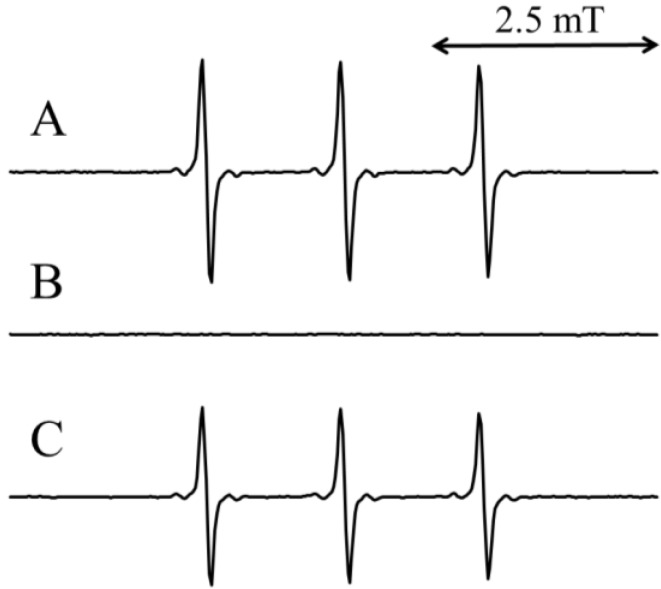
ESR spectra of the standard reaction mixture (III). (**A**) Standard reaction mixture (III); (**B**) Standard reaction mixture (III) with 1 mM CA; (**C**) Standard reaction mixture (III) with 1 mM benzoic acid. Reaction and ESR conditions are as described in the Experimental Section.

We next examined whether CA or benzoic acid inhibited the formation of ^1^O_2_. The trap probe 4-oxo-2,2,6,6-tetramethylpiperidine (4-oxo-TEMP) reacts specifically with ^1^O_2_ to yield the stable nitric oxide radical, 4-oxo-2,2,6,6-tetramethylpiperidinooxy (4-oxo-TEMPO) [36,37], which is characterized by its three-line ESR spectrum. As expected, 4-oxo-TEMPO was detected in the standard reaction mixture (III) (Figure 8A). The addition of 1.0 mM benzoic acid to the standard reaction mixture (III) had no effect on the peak height of 4-oxo-TEMPO, which was 89% ± 4.8% of that obtained with the standard reaction mixture (III) (Figure 8C). In contrast, the addition of 1.0 mM CA to the standard reaction mixture (III), caused the loss of the ESR signals of 4-oxo-TEMPO (Figure 8B). These results suggest that CA, but not benzoic acid, inhibited the formation of ^1^O_2_.

Therefore, CA scavenged ^1^O_2_ directly, in addition to quenching the singlet excited state of FMN. Consistent with this, many studies have reported that flavonoids and polyphenols quench ^1^O_2_ directly. It is possible that the quenching of ^1^O_2_ occurs through an electron transfer reaction or a charge transfer reaction [38,39,40,41,42].

## 3. Experimental Section

### 3.1. Chemicals

4-POBN, oleic acid, ferulic acid, d-(−)-quinic acid, gallic acid, chlorogenic acid, catechol, CA, and *p*-anisic acid were purchased from Tokyo Chemical Industry Co., Ltd. (Tokyo, Japan). FMN, Fe(SO_4_)_2_(NH_4_)_2_, benzoic acid, D_2_O, and 4-oxo-2,2,6,6-tetramethylpiperidine (4-oxo-TEMP) were obtained from Wako Pure Chemical Industries, Ltd. (Osaka, Japan). Noradrenaline was purchased from Nacalai Tesque, Inc. (Kyoto, Japan). 3-Hydroxybenzoic acid and 4-hydroxybenzoic acid were obtained from Kishida Chemical Co., Ltd. (Osaka, Japan). 2-Hydroxybenzoic acid was purchased from Katayama Chemical Co., Ltd. (Osaka, Japan).

### 3.2. Standard Reaction Mixture (I)

The standard D_2_O reaction mixture (I) contained 4.3 mM oleic acid, 25 μM FMN, 160 mM phosphate buffer (pH 7.4), 10 mM cholic acid, 100 mM 4-POBN, and 1 mM Fe(SO_4_)_2_(NH_4_)_2_ in D_2_O in a quartz test tube (100 mm height × 10 mm diameter with 8 mm inside diameter). The standard D_2_O reaction mixtures (I) were prepared as follows: water was removed from standard reaction mixtures lacking 100 mM 4-POBN and 1 mM Fe(SO_4_)_2_(NH_4_)_2_ using a CC-105 centrifugal concentrator (Tomy Seiko Co., Ltd., Tokyo, Japan); D_2_O was then added to reconstitute the reaction mixtures. The standard H_2_O reaction mixture (I) contained the same reagents at the same concentrations, but lacked deuterated molecules. The standard D_2_O (or H_2_O) reaction mixtures (I) without 100 mM 4-POBN and 1 mM Fe(SO_4_)_2_(NH_4_)_2_ were irradiated with 7.8 J/cm^2^ at 436 nm using a REX 250 irradiation system with a LXO436 bandpass filter (Asahi Spectra Co., Tokyo, Japan). After irradiation, 100 mM 4-POBN was added. Reactions were then started by the addition of 1 mM Fe(SO_4_)_2_(NH_4_)_2_, and the mixtures were incubated at 25 °C for 1 min.

### 3.3. Standard Reaction Mixture (II) with CA

The standard D_2_O reaction mixture (II) contained 3.6 mM oleic acid, 20 μM FMN, 166 mM phosphate buffer (pH 7.4), 8.3 mM cholic acid, 83 mM 4-POBN, and 0.8 mM Fe(SO_4_)_2_(NH_4_)_2_ in D_2_O in a quartz test tube (100 mm height × 10 mm diameter with 8 mm inside diameter). The standard D_2_O reaction mixture (II) was prepared, and the reactions were performed as described in Section 3.2.

### 3.4. Standard Reaction Mixture (III)

The standard reaction mixture (III) contained 25 μM FMN, 140 mM phosphate buffer (pH 7.4), and 10 mM 4-oxo-2,2,6,6-tetramethylpiperidine (4-oxo-TEMP) in a quartz test tube (100 mm height × 10 mm diameter with 8 mm inside diameter). The standard reaction mixture (III) was irradiated with 7.8 J/cm^2^ at 436 nm using a REX 250 irradiation system with a LXO436 bandpass filter (Asahi Spectra). After irradiation, ESR measurements were performed by introducing the reaction mixtures into a Teflon tube (1.5 mm diameter with 0.5 mm inside diameter) that passed through the center of the ESR cavity.

### 3.5. ESR Analyses

The ESR measurements were performed on a JES-FR 30 Free Radical Monitor (Jeol Ltd., Tokyo, Japan). The ESR spectrometer settings were as follows: microwave power, 4 mW; modulation width, 0.1 mT; center of magnetic field, 336.300 mT; sweep time, 4 min; sweep width, 10 mT; and time constant, 0.3 s. Hyperfine coupling constants were calculated by splitting of MnO (ΔH_3–4_ = 8.69 mT).

### 3.6. HPLC-ESR Analyses

The HPLC used in the HPLC-ESR consisted of a model 7125 injector (Reodyne, Cotati, CA, USA) and a model L-7100 pump (Hitachi Ltd., Ibaragi, Japan). A semipreparative column (300 mm long × 10 mm i.d.) packed with TSKgel ODS-120T (Tosoh Co., Tokyo, Japan) was used. A flow rate of 2.0 mL/min was used throughout the HPLC-ESR experiments. For the HPLC-ESR, two solvents were employed: solvent A, 50 mM acetic acid; and solvent B, 50 mM acetic acid/acetonitrile (20:80, *v*/*v*). The following isocratic and linear gradients were used: 0–40 min, 100% A to 20% A (linear gradient); 40–60 min, 80% B (isocratic). The eluent was introduced into a model JES-FR30 Free Radical Monitor (Jeol). The ESR spectrometer was connected to the HPLC using a Teflon tube that passed through the center of the ESR cavity. The operating conditions of the ESR spectrometer were as follows: power, 4 mW; modulation width, 0.2 mT; and time constant, 1 s. The magnetic field was fixed at the third peak in the double-triplet ESR spectrum (α^N^ = 1.58 mT and α^Hβ^ = 0.26 mT) of the 4-POBN radical adducts.

### 3.7. Visible Absorption Analyses

Visible absorption spectra were measured using a model UV-160A UV-visible spectrometer (Shimadzu Co., Kyoto, Japan) for the standard reaction mixture (IV) containing 1 mM Fe(SO_4_)_2_(NH_4_)_2_, 2 mM CA, and 40 mM phosphate buffer (pH 7.4). The spectrometer was operated at 400–800 nm, and measurements were obtained at 25 °C. Measurements were obtained in a cuvette with a 10 mm light path. The reference cell contained 40 mM phosphate buffer (pH 7.4).

### 3.8. Fluorescence Measurements

A 650-10S fluorescence spectrophotometer (Hitachi) was used to measure the fluorescence of the standard reaction mixture (V) containing 5.4 mM oleic acid, 31 μM FMN, 182 mM phosphate buffer (pH 7.4), and 5.2 mM cholic acid. The excitation wavelength was set at 436 nm, and emission was recorded from 450 to 700 nm; the width of both slits was 2 nm.

## 4. Conclusions

The formation of 7-carboxyheptyl radicals was inhibited by CA, catechol, gallic acid, chlorogenic acid, ferulic acid, noradrenalin, 2-hydroxybenzoic acid, 3-hydroxybenzoic acid, and 4-hydroxybenzoic acid. These results suggest that a phenol moiety is essential for these inhibitory effects. After the addition of CA, the fluorescence intensity of FMN decreased, suggesting that CA could quench the singlet excited state of FMN. CA also scavenged ^1^O_2_ directly, since the ESR signals of 4-oxo-TEMPO disappeared after the addition of 1.0 mM CA to the standard reaction mixture (III). Therefore, CA may inhibit the formation of 7-carboxyheptyl radicals by scavenging ^1^O_2_ and quenching the singlet excited state of FMN.
